# miR-15b-5p resensitizes colon cancer cells to 5-fluorouracil by promoting apoptosis via the NF-κB/XIAP axis

**DOI:** 10.1038/s41598-017-04172-z

**Published:** 2017-06-23

**Authors:** Ci Zhao, Qi Zhao, Chunhui Zhang, Guangyu Wang, Yuanfei Yao, Xiaoyi Huang, Fei Zhan, Yuanyuan Zhu, Jiaqi Shi, Jianan Chen, Feihu Yan, Yanqiao Zhang

**Affiliations:** 1 Department of Gastrointestinal Medical Oncology, The Affiliated Tumor Hospital of Harbin Medical University, Harbin, 150000 China; 2 Translation Medicine Research and Cooperation Center of Northern China, Heilongjiang Academy of Medical Sciences, Harbin, 150000 China; 3 Department of Biotherapy, The Affiliated Tumor Hospital of Harbin Medical University, Harbin, 150000 China

## Abstract

Drug resistance, which is closely correlated with an imbalance in apoptosis, endows colorectal cancer (CRC) with enhanced progression capacity irrespective of the treatment with therapeutics. We report that miR-15b-5p is a tumor suppressor whose level is globally decreased in CRC cells and tissues. Over-expression of miR-15b-5p not only promoted 5-fluorouracil (5-FU)-induced cellular apoptosis but also reversed the chemoresistance of 5-FU *in vitro* and *in vivo*. As a key mediator of inflammation-induced cancer, miR-15b-5p enhances these therapeutic effects are mainly attributed to targeting of the NF-κB signaling pathway through negative regulation of NF-κB1 and one of its kinase complexes IKK-α. miR-15b-5p mediates NF-ĸB regulation by targeting the anti-apoptosis protein XIAP *in vitro*. Together, these results establish an axis of miR-15b-mediated apoptosis regulation, which reverses chemoresistance and suppresses CRC progression. These findings suggest that miR-15b-5p may be a potential agent for CRC treatment, particularly for 5-FU-resistant CRC.

## Introduction

For over 50 years, 5-fluorouracil (5-FU) has been used as the first-line chemotherapeutic agent for colorectal cancer (CRC)^1^; however, the response rate of advanced CRC to 5-FU is only 10–15%^2^. Treatment with 5-FU in combination with oxaliplatin or irinotecan has improved the response rate of advanced CRC patients to 40–50%^3, 4^. A critical factor that inevitably limits the efficacy of chemotherapy is drug resistance, which can be classified into intrinsic and acquired resistance. Not surprisingly, acquired resistance is more frequent during the course of anticancer drug treatment including chemotherapy and targeted therapies^5, 6^; many cancer patients who initially respond well to chemotherapy gradually exhibit decreased sensitivity to the specific chemotherapeutic. This acquired resistance may be attributed to long-term drug exposure, resulting in the development of mutations or adaptive processes; however, the mechanisms underlying such chemoresistance remain to be fully elucidated.

Epidemiological data demonstrate a strong connection between chronic inflammation and cancer development and suggest that up to 25% of all cancers, especially colorectal cancer, result from chronic infection or other types of chronic inflammation^7^. Many clinical trials have reported that non-steroidal, anti-inflammatory drugs (NSAIDs) provide protection against colon adenomas, thereby acting as protectors against CRC when administered long-term^8, 9^. Chronic inflammation can become oncogenic by various mechanisms including the induction of genomic instability, increased angiogenesis, altered genomic epigenetic state, and increased cell proliferation^10^. Key mediators of inflammation-induced cancers include, among others, nuclear factor kappa B (NF-κB) and specific microRNAs^11–13^. An improved understanding of the interconnections between miRNA, inflammation, and cancer may thus provide novel therapeutic strategies. Moreover, in many solid tumors, especially in CRC, constitutive activation of NF-κB has been observed^14^, where NF-κB acts as a transcription factor that contributes to the progression of CRC by regulating the expression of diverse target genes involved not only in inflammation but also in apoptosis (*XIAP, Survivin, Bcl-2*, *Bcl-xl*, and others). The transcription factor also provides a survival mechanism by up-regulating anti-apoptotic genes, and accordingly, NF-κB may be a causative factor in drug resistance^15^.

miR-15b-5p is negatively associated with apoptosis and drug resistance and could be a key inflammatory mediator of the NF-κB family in CRC patients. The aim of this study was to assess the expression patterns of miR-15b-5p in multiple CRC cell lines as well as in CRC tissues, and then to investigate a potential correlation between miR-15b-5p and 5-FU chemosensitivity of CRC using two cell lines and animal models.

## Results

### miR-15b-5p expression is decreased in CRC cell lines and patient specimens

**Figure 1:**
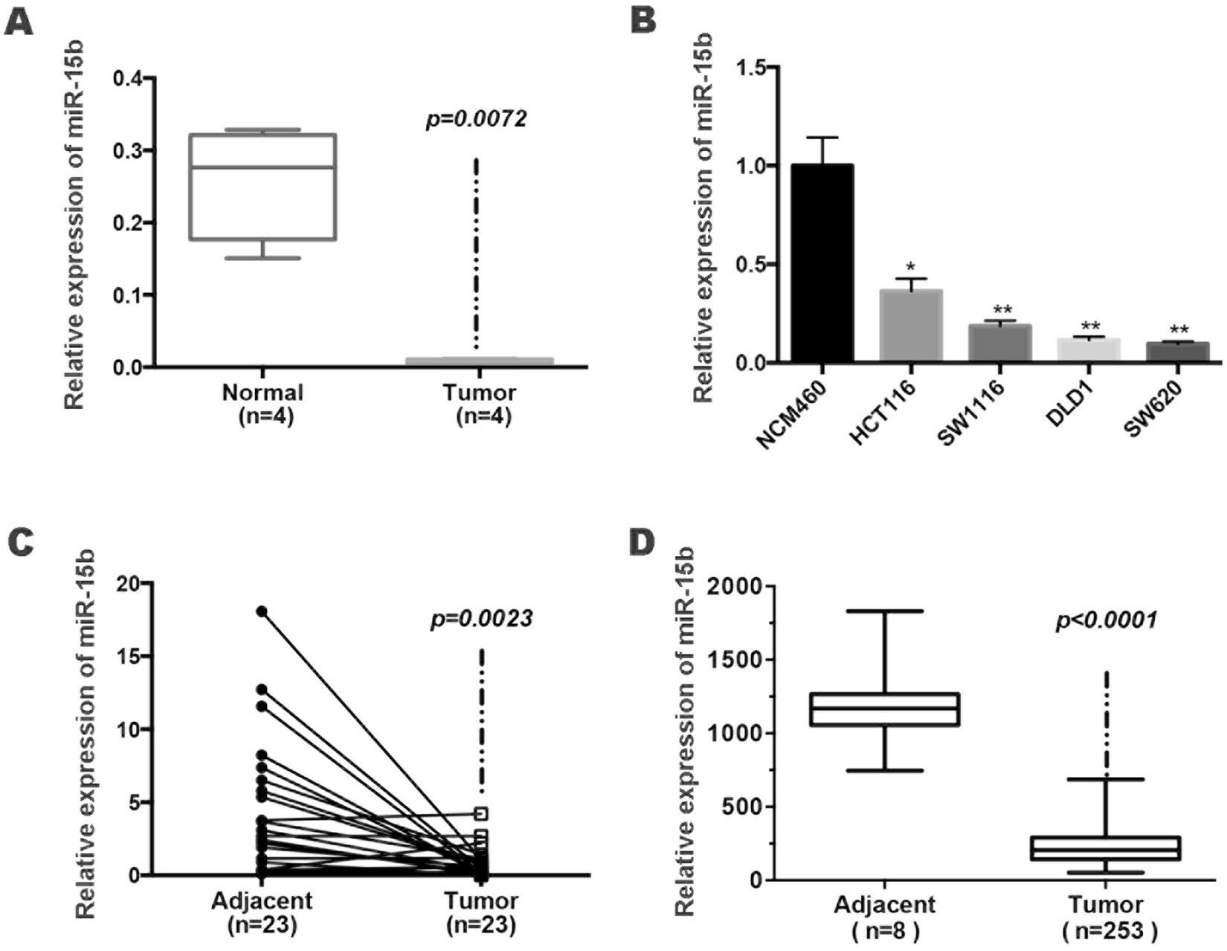
miR-15b-5p was downregulated in CRC. (**A**) Real-time PCR confirmation of miRNA microarray results was performed in four paired tumor and normal tissues in CAC mice. (**B**) Determination of miR-15b-5p expression levels in four colon cell lines and NCM460 cells (*p < 0.05, **p < 0.01, ***p < 0.001). (**C**) Downregulation of miR-15b-5p in primary colorectal cancer tissue compared to the adjacent normal tissue (paired Student’s *t*-test). (**D**) miR-15b-5p expression levels decreased significantly in primary colorectal cancer tissue compared to normal tissue via the TCGA data.

To investigate the roles of miRNAs in colorectal cancer, miRNA microarray analysis was performed in colitis-associated colon cancer (CAC) models^16^. Among the differentially expressed miRNAs, miR-15b-5p was stably down-regulated in the cancer tissues (data not shown). To validate the microarray results, the significant downregulation of miR-15b-5p was confirmed by real-time PCR in four mouse models of CAC (Fig. 1A) as well as in four colon carcinoma cell lines including SW620 (~0.10 fold), DLD1 (~0.12 fold), SW1116 (~0.19 fold), and HCT116 (~0.36 fold). Normal NCM460 cells served as a control (Fig. 1B). In addition to the animal model and cell lines, analysis of clinical samples revealed that miR-15b-5p expression was lower in tumor tissues than in adjacent normal colon tissues (Fig. 1C). Finally, the downregulation of miR-15b-5p in CRC patients’ tissues was further validated using data collected from different study groups in the Cancer Genome Atlas (TCGA) Data Portal (Fig. 1D).

### miR-15b-5p enhances the sensitivity of colon cancer cells to 5-FU

Chemotherapy regimens for the treatment of CRC are primarily fluorouracil (5-FU)-based, where 5-FU is generally combined with oxaliplatin and leucovorin. To investigate the potential role of miR-15b-5p in chemosensitivity in CRC, SW620 and HCT116 colon carcinoma cells were treated with different concentrations of 5-FU for 48 h. A cell viability assay showed that the half maximal inhibitory concentration (IC_50_) of 5-FU in SW620 cells (444.7 µg/mL) was significantly higher than that in HCT116 cells (56.36 µg/mL; Figure S1A). Interestingly, miR-15b-5p expression levels were found to be inversely proportional to the chemotherapeutic sensitivity of the two colon carcinoma cell lines. SW620 cells were previously reported to be resistant to 5-FU^17^, thus suggesting that miR-15b-5p acts as a mediator of 5-FU sensitivity in these cells.

**Figure 2:**
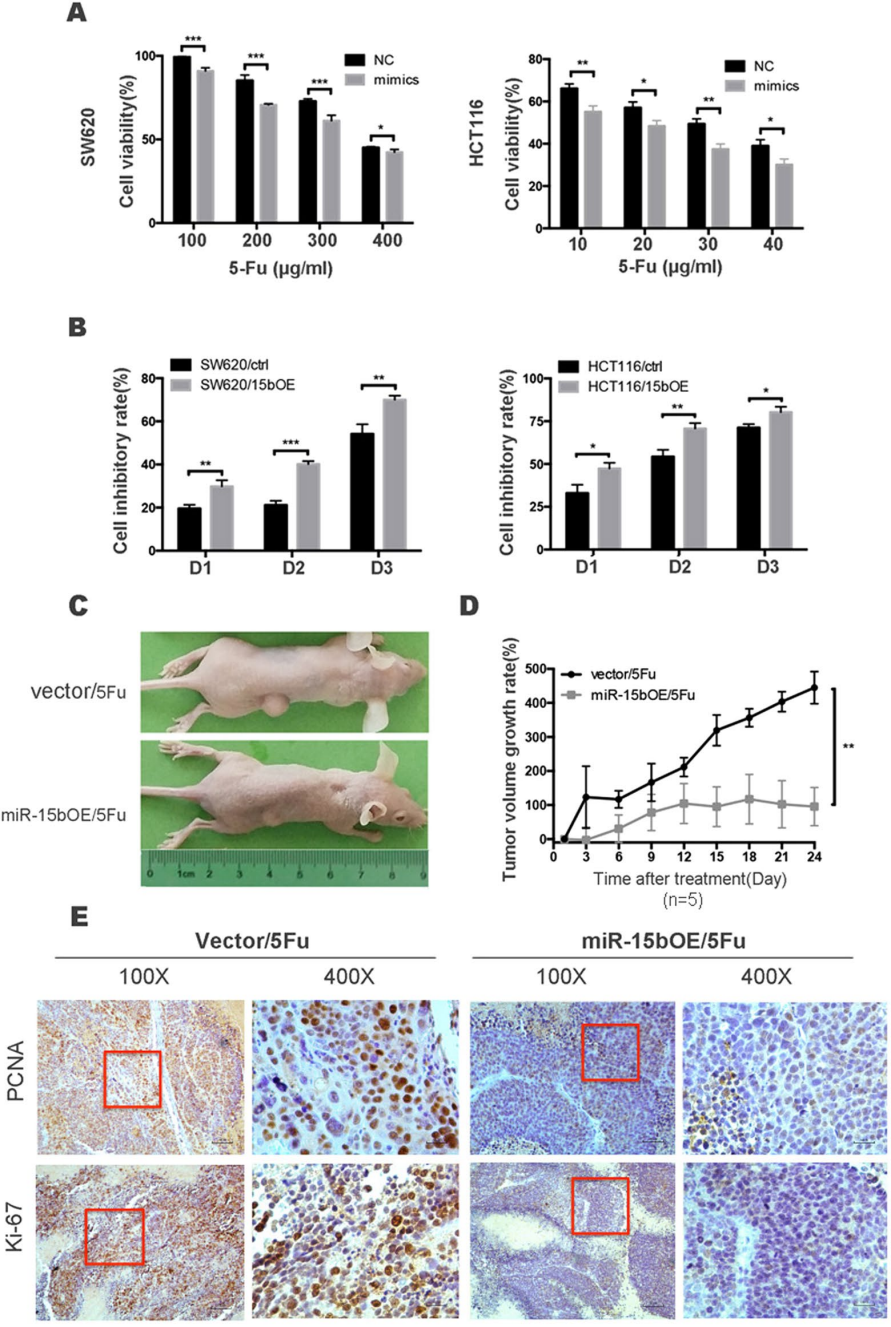
miR-15b-5p enhances chemotherapeutic sensitivity in colon cancer cells *in vitro* and *in vivo*. (**A**) The miR-15b-5p-transfected cells treated with 5-FU as the dosage increased for 48 hours, the cell viability as observed by luminescent cell viability assay in HCT116 and SW620 cells. (**B**) CCK-8 assays were performed on miR-15b-5p overexpressing (miR-15bOE) and vector control cells with an appropriate dosage of 5-FU of 300 μg/mL for SW620 and 30 μg/mL for HCT116, and the cell inhibition rate at each time point is shown as a percentage (*p < 0.05, **p < 0.01, ***p < 0.001). All data are representative of 3 independent experiments. (**C**) SW620/miR-15b OE or SW620/vector cells were subcutaneously inoculated into the dorsal right flanks of 5-week-old nude mice, then the tumor size was observed after 5-FU (20 mg/kg) by the intraperitoneal injection. (**D**) Tumor volume growth rate was calculated at the indicated intervals. N = 5, *p* = 0.0054 (**E**) Representative results of the immunohistochemistry assays showing the effect of miR-15b-5p expression on proliferative abilities *in vivo*. Representative image magnification: 100x and 400x.

Next, the extent to which miR-15b-5p affects chemosensitivity to 5-FU in colon carcinoma cell lines was investigated. A luminescent cell viability assay showed that transfection of HCT116 and SW620 cells with miR-15b-5p mimics significantly decreased the 5-FU IC_50_ in these cell lines compared with the same cells lines transfected with non-specific miRNAs (Figure S1B). After 48 hours of treatment, the miR-15b-5p mimics induced an increase in sensitivity to the chemotherapeutic agents at four different concentrations (100, 200, 300, and 400 µg/mL), with more miR-15b-5p mimic-transfected cells than non-specific miRNA-transfected cells undergoing apoptosis after the treatment (Fig. 2A). To determine whether the effect of altered miR-15b-5p expression on 5-FU sensitivity is time dependent, a miR-15b-5p overexpression (miR-15bOE) model was further established using HCT116 and SW620 cells, and was subsequently used for chemosensitivity assays. As shown in Figure S1C, miR-15b-5p expression was elevated by ~10–12 fold in the miR-15bOE cells relative to the vector control cells. Induced miR-15b-5p expression was furthermore shown to significantly enhance the sensitivity of both HCT116 and SW620 cells to 5-FU after 24, 48, and 72 hours of treatment (Fig. 2B). These findings indicate that miR-15b-5p plays an important role in 5-FU resistance *in vitro*.

Next, the potential sensitization to 5-FU by miR-15b-5p was assessed in a xenograft model. Xenograft tumors were generated by subcutaneously injecting 4 × 10^6^ SW620/miR-15bOE or SW620/vector cells into immunodeficient mice. After 15 days, mice were injected with 20 mg/kg 5-FU, and tumor size was measured every 2 days. During 5-FU treatment, SW620/miR-15bOE tumors in mice were found to be significantly decreased in size relative to those in the control group (Fig. 2C and D). Immunohistochemistry (IHC) studies on tumor tissues demonstrated that the expression levels of the proliferation markers PCNA and Ki-67 were significantly lower in the SW620/miR-15bOE tumors than in the control (Fig. 2E). These findings suggest that miR-15b-5p can reverse the tolerance of 5-FU-resistant tumors by inhibiting cell expansion.

### miR-15b-5p alters colon cancer chemosensitivity by enhancing cell apoptosis

**Figure 3:**
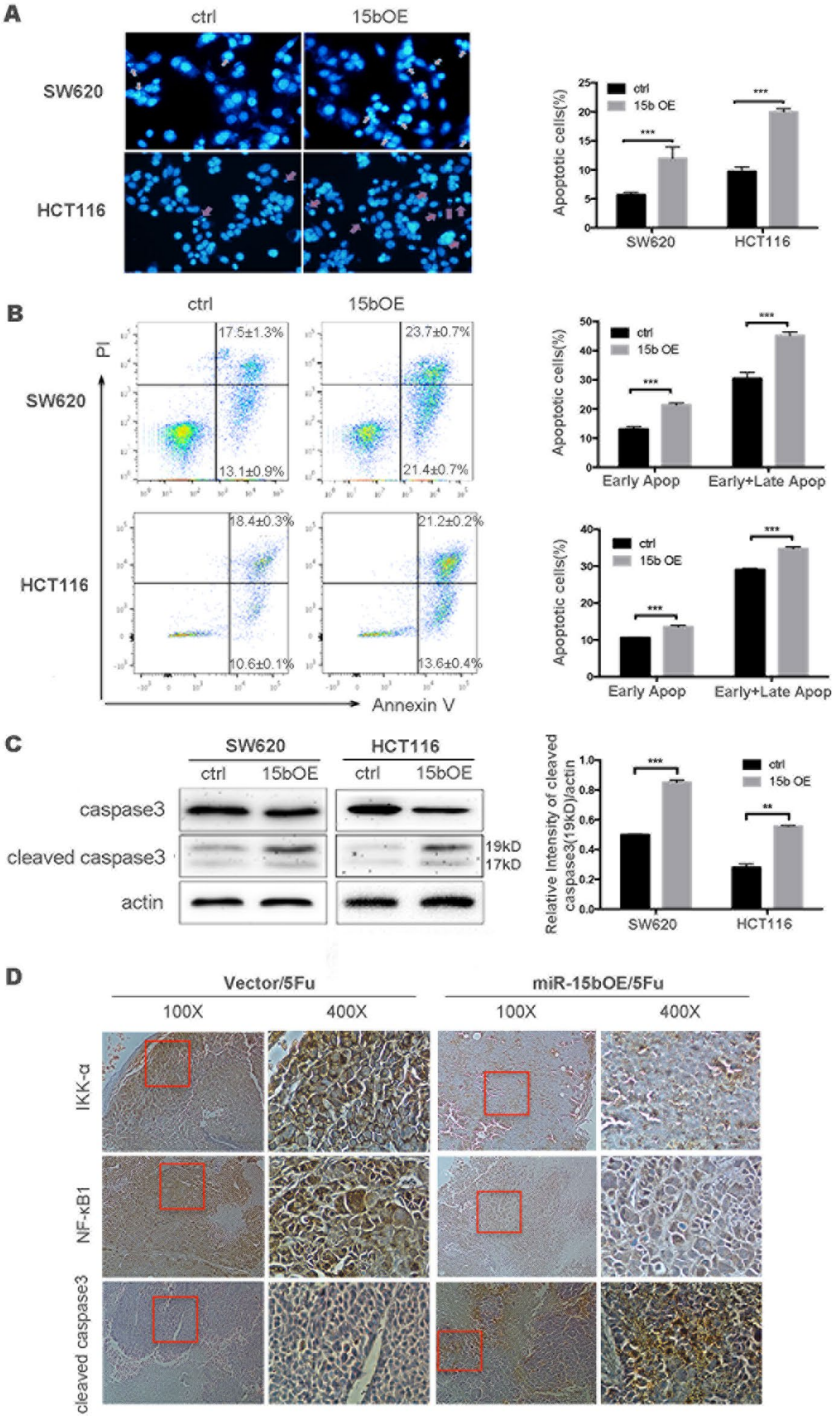
Overexpression of miR-15b-5p promotes apoptosis induced by 5-FU. (**A**) Apoptosis was analyzed by DAPI staining, after 5-FU-induced miR-15bOE and control cells shown representative image of apoptotic cells; and counting the percentage of apoptotic cells; Red marker pointed the apoptotic cell. (**B**) Flow cytometry was used to evaluate PI/Annexin V staining. miR-15b-5p increased 5FU-induced apoptosis in SW620 or HCT116 cells. The percentage of cells in the early or early plus late apoptotic stage was increased in miR-15bOE cells compared with control cells after the cells were exposed to 5-FU for 48 h. (**C**) Cleaved caspase3 protein detected by western blotting. The levels of cleaved caspase3 increased in miR-15bOE cells treated with 5-FU. Semi-quantitative data from densitometric analysis of cleaved caspase3 are presented as the relative ratio of each protein to actin (*p < 0.05, **p < 0.01, ***p < 0.001). (**D**) Representative images of the IHC examination of IKK-α, NF-ĸB1, and cleaved caspase3 in mouse tumors from miR-15b OE cells and vector mice after 5-FU treatment.

Because reduced apoptosis is an important factor for unlimited expansion and chemotherapeutic resistance of cancer cells^18^, the apoptotic capacity of 5-FU-induced miR-15bOE/vector colon carcinoma cells was assessed. After 48 hours of 5-FU treatment, DAPI staining showed that forced expression of miR-15b-5p resulted in a ~2-fold increase in the number of apoptotic cells in HCT116 cells (20.0 ± 0.6%) relative to the control cells (9.7 ± 0.8%). Similar results were observed for SW620 cells, with 12.0 ± 1.9% for miR-15bOE *vs*. 5.7 ± 0.4% for control cells (Fig. 3A). The percentage of apoptotic cells was also measured by flow cytometry, which revealed that the percentage of early and late apoptotic cells was prominently increased in miR-15bOE cells compared with control cells (45.1 ± 1.3% *vs*. 30.5 ± 2.0%, respectively, in SW620 cells; 34.8 ± 0.5% *vs*. 29.0 ± 0.4%, respectively, in HCT116 cells) (Fig. 3B). These findings suggest that miR-15b-5p overexpression enhanced the sensitivity of cells to 5-FU, with more cells undergoing apoptosis after the treatment. Western blot analysis revealed a 2.0-fold increase in the level of cleaved caspase 3 in miR-15b-5p-overexpressing HCT116 cells compared with control HCT116 cells (Figs 3C and S2B), accompanied by a decline of caspase 3 (Figure S2A). Similar results were obtained for SW620 cells (Fig. 3C). The *in vitro* results reported here were further supported by *in vivo* data: cleaved caspase 3 levels were significantly increased in SW620/miR-15bOE tumors relative to SW620/vector tumors during treatment with 5-FU (Fig. 3D), accompanied by significant reductions in the levels of NF-κB1 and IKK-α, two key modulators in inflammation and cell apoptosis. These results collectively indicate that miR-15b-5p sensitizes cancer cells to apoptosis via the NF-κB pathway.

### miR-15b-5p targets *NF-κB1* and *IKK-α* and suppresses NF-κB-dependent survival proteins in colon cancer cells

**Figure 4:**
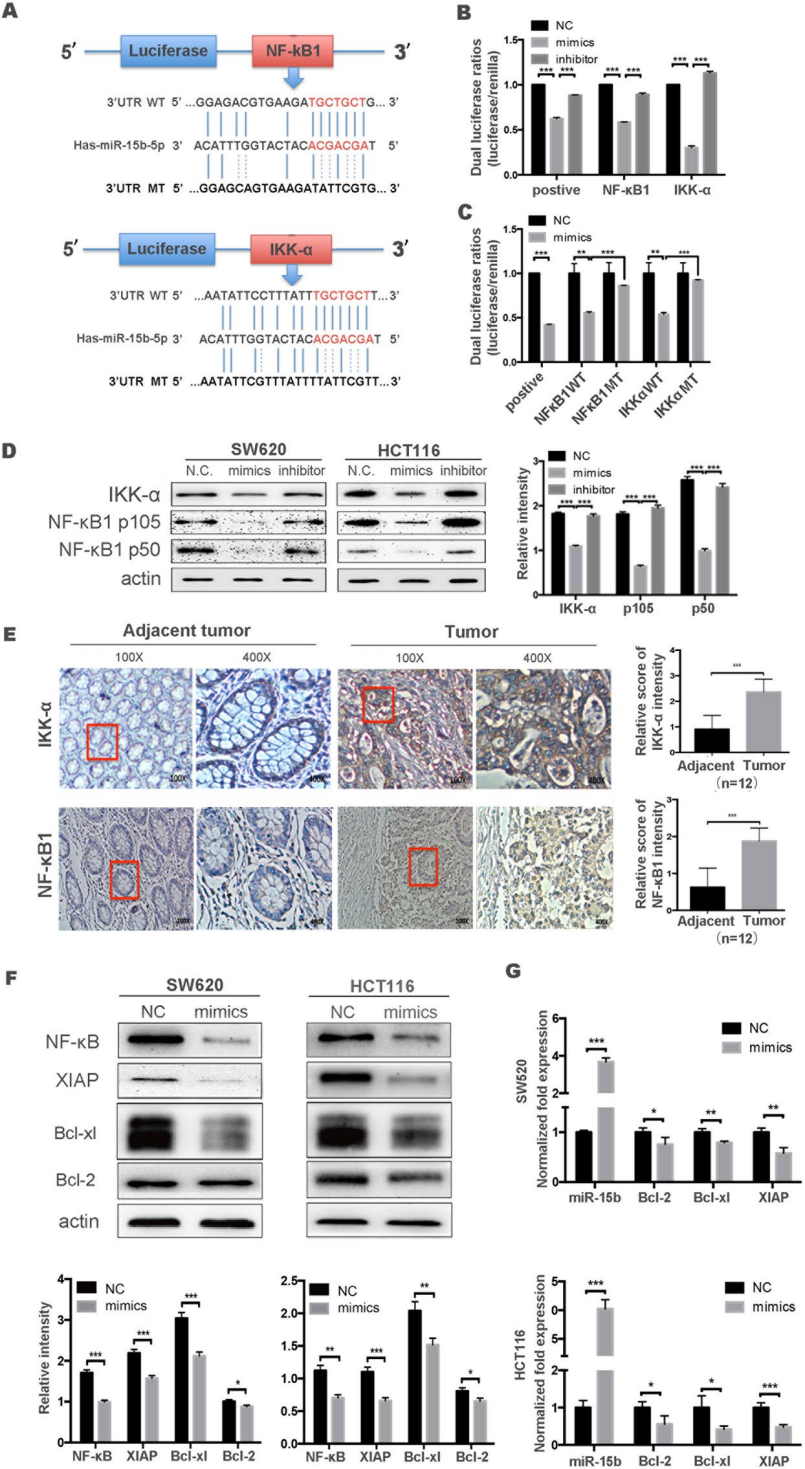
NF-ĸB1 and IKK-α are targets of miR-15b-5p in colon cancer cells. (**A**) Schematic representation of the miR-15b-5p putative binding sites in the 3-UTR of *NF-ĸB1* or *IKK-α* mRNA and the mutations introduced into the 3-UTR regions. (**B**) Wild-type (WT) or mutated type (MT) *NF-ĸB1* or *IKK-α* reporter constructs were co-transfected with miR-15b-5p mimics or NC in HEK293T cells. (**C**) miR-15b-5p mimic or inhibitor was co-transfected with the pMIR-NFĸB1-3′UTR or pMIR-IKKα-3′UTR vector in HEK293T cells. The relative luciferase activity was measured. (**D**) NF-ĸB1 and IKK-α protein expression after transfection of miR-15b-5p mimic or inhibitor in SW620 and HCT116 cells. Cells were evaluated at 48 h after transfection by western blotting. Semi-quantitative data from densitometric analysis were presented as the relative ratio of each protein to actin. (**E**) Representative images of the IHC staining of NF-ĸB1 or IKK-α in 12 colorectal cancer patients. (**G**) Western blot analysis of anti-apoptotic XIAP, Bcl-xl, and Bcl-2 protein levels in SW620 or HCT116 cells at 48 h after transfection with the miR-15b-5p mimic or a negative control. Actin was used as an internal control. (**F**) RT-qPCR analysis of relative mRNA expression level in cells at 24 h after transfection with the miR-15b-5p mimic or negative control (*p < 0.05, **p < 0.01, ***p < 0.001).

Using online databases (TargetScan and MicroCosm), *NF-ĸB1* and *IKK-α* the genes encoding NF-ĸB1 and IKK-α, which are both associated with the NF-κB pathway, were identified as potential targets of miR-15b-5p. Putative binding sites for miR-15b-5p were also identified in the 3′-UTRs of *NF-κB1* and *IKK-α*. The seed sequences of hsa-miR-15b-5p have 7 nucleotides exactly complementary to the nucleotides at 272–278 and 967–973 bp upstream of the start codons of *NF-κB1* and *IKK-α* mRNA, respectively. To validate these putative binding sites, the WT and MT 3′-UTRs of *NF-κB1* and *IKK-α* were individually cloned into the pMIR-reporter vector and direct binding between miR-15b-5p and the target gene transcripts was then assessed by dual luciferase reporter assays (Fig. 4A). When miR-15b-5p mimics were co-transfected with the WT and MT pMIR-NFκB1-3′-UTR or the pMIR-IKKα-3′-UTR vector in HEK293T cells, luciferase assays showed that ectopic expression of miR-15b-5p significantly decreased the activity of the WT but not that of the MT (Fig. 4B). When miR-15b-5p mimics or inhibitors were co-transfected with the pMIR-report vectors, the luciferase activity of mimics significantly decreased compared with that in cells co-transfected with the negative control (NC) or inhibitors (Fig. 4C).

Next, the effects of miR-15b-5p overexpression on the endogenous levels of NF-κB1 or IKK-α were assessed. Western blot analysis showed that the endogenous protein levels of NF-κB1 or IKK-α were substantially decreased by miR-15b-5p overexpression and that expression was rescued when colon carcinoma cells were transfected with miR-15b-5p inhibitors (Fig. 4D). The inhibition effect was dose-dependent (Figure S3A). After transient transfection of miR-15b-5p mimics, the mRNA levels of IKK-α furthermore exhibited the same trend observed at the protein level, indicating that miR-15b-5p represses the expression of IKK-α by specifically binding with and subsequently inducing the degradation of *IKK-α* mRNA. In contrast to *IKK-α* mRNA, the levels of the transcription product of NF-κB1 were barely altered following miR-15b-5p mimic transfection (Figure S3C), which suggests that miR-15b-5p primarily reduces *NF-κB1* levels by selectively inhibiting the translation of *NF-κB1* mRNA and not by mRNA degradation.

In addition to the above-mentioned *in vitro* experiments, *in vivo* experiments were performed to validate the association between miR-15b-5p and NF-κB1 or IKK-α expression levels in 12 colorectal cancer specimens. As anticipated, the expression of miR-15b-5p was always conversely associated with NF-κB1 or IKK-α expression in these tissues (Figs 4E and S3D). Moreover, miR-15b-5p targeting of *NF-κB1* or *IKK-α* was evidenced by the finding that both the protein (Fig. 4F) and mRNA levels (Fig. 4G) of the various downstream anti-apoptotic targets of the NF-κB pathway such as XIAP, Bcl-xl, and Bcl-2 were significantly decreased concomitantly with NF-κB when SW620 and HCT116 cells were transiently induced to over-express miR-15b-5p. Collectively, these data indicate that miR-15b-5p represses NF-κB1 and IKK-α expression due to the decreased levels of NF-κB.

### Overexpression of XIAP decreases the inhibitory effects of miR-15b-5p on drug resistance in colon cancer cells

**Figure 5:**
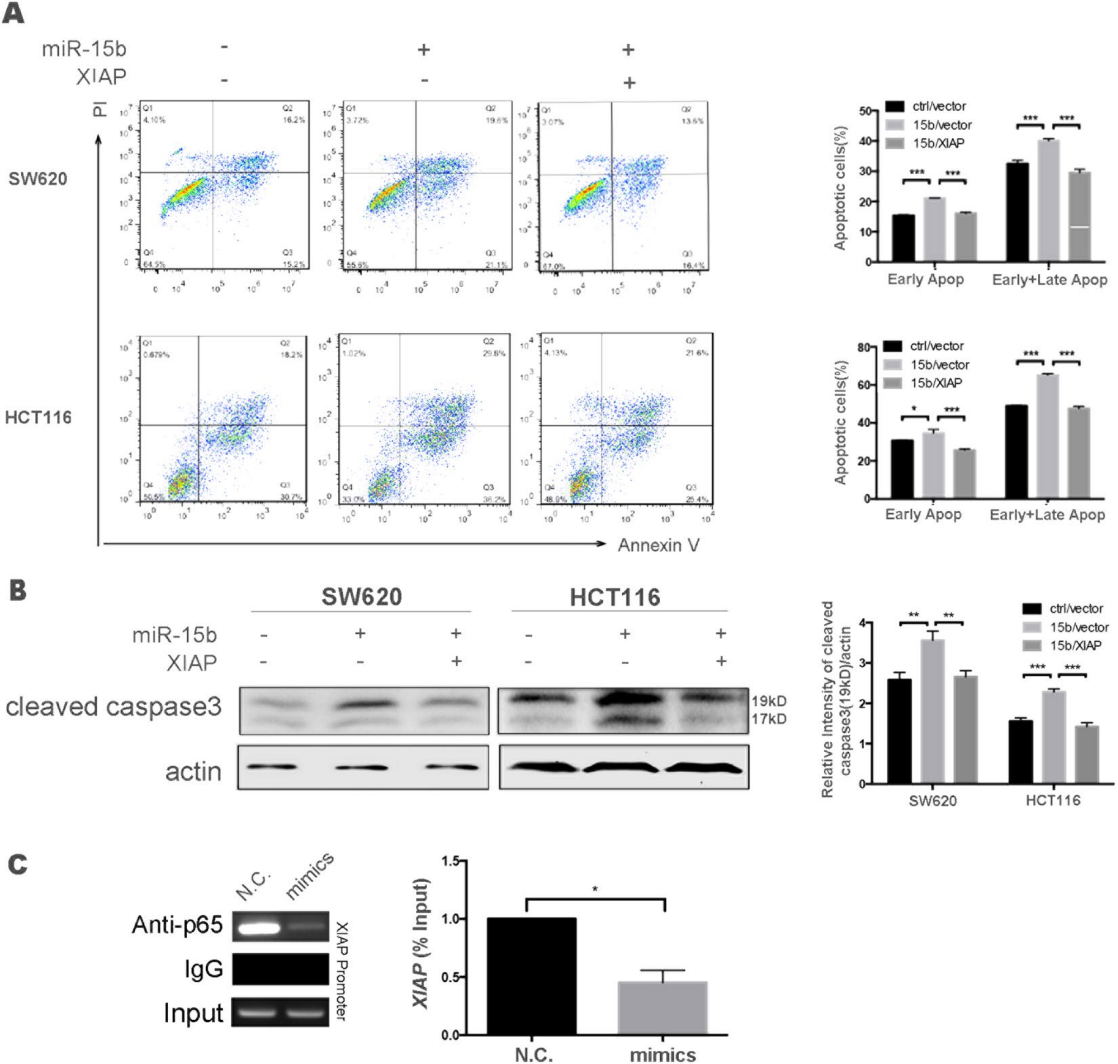
Overexpression of XIAP abolishes miR-15b-5p-induced apoptosis of colon cancer cells and drug sensitivity. (**A**) Flow cytometry analysis showed that the reintroduction of XIAP reduced the sensitivity of miR-15b-5p to 5-FU to apoptosis. (**B**) Western blot analysis of cleaved caspase 3 protein levels after XIAP or miR-15b-5p were co-transfected into HCT116 and SW620 cells. (**C**) ChIP assay was performed by using HEK293T cells. Chromatin was immunoprecipitated by using p65 specific antibody. Regular PCR was carried out by using primers of *XIAP* promoter.

Because XIAP overexpression is often observed in several human cancers and is closely correlated with chemotherapy resistance, the potential for elevated XIAP expression to abolish the pro-apoptotic function of miR-15b-5p in colon cancer was investigated in this study. A eukaryotic expression vector (PCMV-C1-EGFP-XIAP) was constructed, and its expression in HEK293T cells was confirmed by western blot (Figure S4A). Next, the expression vector, whose expression pattern of XIAP was confirmed by western blot (Figure S4B), was transfected into miR-15b OE or vector control cells, after which 5-FU-induced apoptosis was evaluated in these cells by flow cytometry. As shown in Fig. 5A, the HCT116 cells in which miR-15b-5p overexpression was induced exhibited significantly elevated rates of early and late apoptosis after treatment with 5-FU for 48 hours (miR-15b *vs*. control; 65.2 ± 0.8% *vs*. 49.1 ± 0.3%, *p* < 0.001). In contrast, HCT116 cells induced to overexpress both miR-15b-5p and XIAP remained at baseline levels of apoptosis after treatment with 5-FU (miR-15b + XIAP *vs*. control; 47.6 ± 1.1% *vs*. 49.1 ± 0.3%, *p* > 0.05). Similar results were observed in SW620 cells (Fig. 5A). In the miR-15b-5p/XIAP dual-overexpressing HCT116 cells, expression of cleaved caspase 3, the key initiator responsible for promoting cell apoptosis, was also constrained to a low level comparable to that in the control cells (Fig. 5B). To investigate how miR-15b-5p regulates the expression of XIAP, ChIP assays were performed in HEK293T cells by using p65 specific antibody. As shown in Fig. 5C, p65 bound with XIAP promoter, and miR-15b-5p decreased the expression of XIAP through the NF-κB pathway. These findings confirm that XIAP plays key roles in miR-15b-5p-enhanced 5-FU sensitization and thus that XIAP is a potential candidate for reversing drug resistance and suppressing colorectal cancer progression.

## Discussion

miRNAs have various functions in tumorigenesis, which are achieved via modulation of target genes. Accordingly, miRNAs serve as biomarkers or therapeutic targets for the treatment of cancer^19^. Particularly in drug-resistant tumors, the most determinant property for miRNA efficacy against chemotherapy resistance is the ability of the miRNAs to induce cell apoptosis. In this regard, this study focused on the role of miR-15b-5p, which we identified as being aberrantly expressed in colon cancer using the CAC model, in the chemoresistance of colon cancer.

In the canonical NF-κB pathway, NF-κB is a protein dimer consisting of p50 and Rel protein p65, and is activated by the IκB-kinase (IKK) complex^20^ before binding to κB sites in the genome, thereby regulating the expression of proinflammatory genes, immune response-related genes, and other biological processes^21^. And Ruusalepp *et al*. demonstrated that nfκb1^−/−^ mice displayed reduced inflammatory gene expression and enhanced neointimal formation in response to ligation. Therefore NF-κB1 is an important regulator for inflammatory genes expression and thus in turn limiting vascular healing via pro-inflammatory activity^22^. In addition, this study is based on inflammation-induced colorectal cancer model data, which is from inflammation, adenoma to adenocarcinoma, we are more concerned about inflammation-related factors such as NF-κB1 and its related factors. So we researched the role of miR-15b-5p in the NF-κB pathway. In principle, two mechanisms are involved in miRNA-mediated target gene silencing: (1) selective guidance of the complimentary mRNA to degradation and (2) translation depression of the target^23^. In this study, the core sequence of miR-15b-5p was complementary to the 3′-UTR of *NF-κB1*(*p105/50*). The p105 precursor is constitutively processed into active p50 by proteasomes, including KPC1 ubiquitin E3 ligase; co-translational proteasomal processing; and 20 s proteasomal processing^24–26^. Because there is no predicted consequential pairing on the 3′-UTR of these proteasome genes, it is unlikely that miR-15b-5p has an effect on the post-translational modification of p105. Accordingly, our focus in this study was on the translation of NF-κB1. miR-15b-5p was shown to inhibit both p105 and p50 protein, whereas the corresponding mRNA levels of these proteins decreased only slightly upon miR-15b-5p overexpression. These results indicate that NF-κB1 translation was inhibited by miR-15b-5p.

Next, miR-15b-5p overexpression was shown to also lead to a significant reduction in p65, whose 3′-UTR lacks a pairing region for miR-15b-5p (Figure S3B). Further investigation on the upstream of p65 revealed IKK-α as another target of miR-15b-5p, where IKK-α is one of the catalytic subunits of the IκB-kinase (IKK) complex, which is stringently stimulated in the NF-κB canonical pathway^20^. IKK-α plays a pivotal role in regulating the entire pathway, which can induce the DNA binding activity of NF-κB/p65^27, 28^ and is required for generation of transcriptionally competent NF-κB, ultimately resulting in increased expression of NF-κB-dependent genes^29^. Our study demonstrated that miR-15b-5p specifically binds to *IKK-α* mRNA and decreases the expression of *IKK-α* at the mRNA and protein levels simultaneously. Mechanically, the mRNA of *IKK-α* is therefore degraded via binding with miR-15b-5p. In response to the degradation of *IKK-α* mRNA, the expression of the downstream p65 gene was further reduced consistently. Collectively, the aforementioned results indicate that both the p65 and p50 dimers of NF-κB are inhibited by miR-15b-5p. The expression of NF-κB-dependent anti-apoptotic genes (*XIAP, Bcl-xl*, and *Bcl-2*) was also shown to be downregulated, suggesting that miR-15b-5p negatively regulates NF-κB1 and IKK-α, resulting in the suppression of NF-κB-dependent survival proteins in colorectal cancer. The detailed mechanisms by which this activation and altered activation occur remain to be investigated.

**Figure 6:**
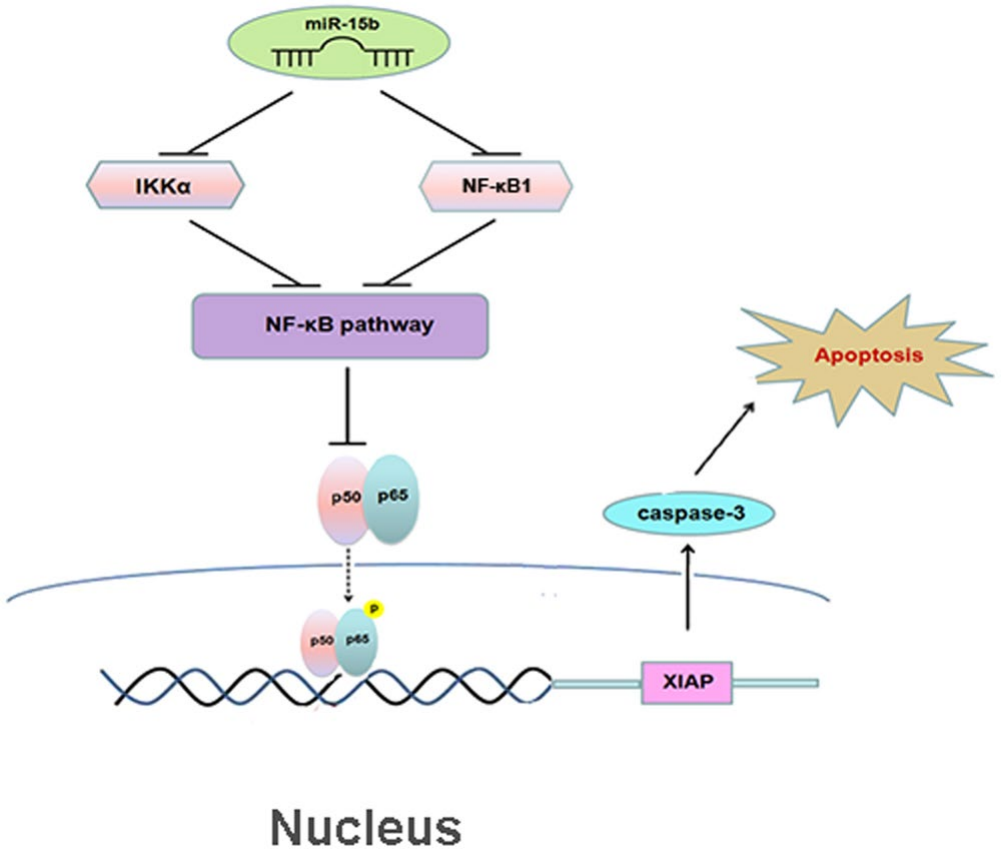
The schematic diagram of possible molecular mechanism of miR-15b-5p-induced apoptosis in CRC cells.

Chemoresistance associated with 5-FU is a complex and multifactorial process, which involved several mechanisms, and the key point is the imbalance of apoptosis^30^. NF-κB provides a survival mechanism by up-regulating anti-apoptotic genes and thereby represents a major causative factor for drug resistance^15^. Among the factors downstream of the NF-κB pathway, anti-apoptotic XIAP acts as a central regulator of apoptosis by inhibiting the caspase cascade, specifically by directly inhibiting active caspases -3, -7, and -9 and thus functioning as an endogenous inhibitor of caspase-dependent apoptotic cell death^31–34^. Overexpression of XIAP in various cancers has been reported to be associated with chemoresistance, poor prognosis, and progression of disease^35, 36^. Our study clearly demonstrates that after colon cells are transiently transfected with miR-15b-5p mimics and the NF-κB pathway is suppressed, XIAP expression is dramatically down-regulated at both the protein and mRNA levels. Bioinformatics analysis did not identify a potential target site for miR-15b-5p in the 3′-UTR of *XIAP*; but ChIP assays determined that XIAP suppression by miR-15b-5p may be mediated by inhibition of the NF-κB pathway rather than by targeting by miR-15b-5p. This notion is further supported by the finding that cleaved caspase-3 levels were also downregulated immediately after 5-FU treatment. Co-transfection with miR-15b-5p and *XIAP* expression vectors resulted in decreased apoptosis rates compared with the rate in cells transfected with miR-15b-5p alone, but rates similar to those observed for vector control-transfected cells. The expression levels of cleaved caspase-3 exhibited the same tendency as the apoptosis rates. Thus, it can be inferred that XIAP is a target of miR-15b-5p-mediated enhancement of drug sensitivity and programmed cell death. Our study provides new insight into the mechanism of miR-15b-5p-mediated drug resistance and apoptosis (Fig. 6) that differs from a previously published mechanism^37^.

In conclusion, our results show that miR-15b-5p is down-regulated in CRC cells and tissues and that the inhibitory effects of miR-15b-5p on cell apoptosis and enhancement of drug sensitivity are mediated by the down-regulation of its NF-κB1 and IKK-α targets. These findings reveal a potential mechanism underlying the tumor-suppressing role of miR-15b-5p and suggest that miR-15b-5p is a potentially useful marker and therapeutic target for colon cancer.

## Experimental Procedures

### Clinical samples

The present study included 23 CRC tissues and their adjacent normal mucosa, which were resected at the Department of Gastrointestinal Surgical Oncology of The Affiliated Tumor Hospital of Harbin Medical University in 2013. None of the patients received preoperative treatment such as irradiation or chemotherapy. Written informed consent was obtained from the patients, in accordance with institutional guidelines, before sample collection, and the study was approved by the Committees for the Ethical Review of Research at Harbin Medical University. The methods were performed in accordance with the approved guidelines. All patients had a histological diagnosis of colorectal cancer and received radical resection. None of the patients included in the study had received neoadjuvant therapy before surgery. The matching adjacent noncancerous tissue and primary colorectal cancer tissue were retrieved from the Tissue Bank of Harbin Medical University Affiliated Tumor Hospital.

### Cell lines and Transfection

The normal human colonic mucosa cell line NCM460 and the human colorectal carcinoma cell lines SW620, HCT116, DLD1, and SW1116 were provided by the Shanghai Institute of Cell Biology. The normal and carcinoma cell lines were maintained in RPMI-1640 and Dulbecco’s modified Eagle medium, respectively, containing 10% FBS. The cells were cultured in a humidified 5% CO_2_ incubator at 37 °C. Human mature hsa-miR-15b-5p mimics and a negative control were designed and synthesized by Dharmacon (Lafayette, CO) and were transfected using X-tremeGENE HP DNA transfection reagent (Roche, CA, USA.). In transient transfection, cells were harvested at 24 or 48 hours after transfection. For selection of stably transfected cells, puromycin (0.85 μg/mL) was added to the growth medium 48 hours after transfection, and individual puromycin-resistant colonies were picked and expanded two weeks later.

### Plasmid construction

Lentiviral vector harboring miR-15b-5p (miR-15b OE) sequence was constructed by PCR amplify the pri-miR-15b sequence using the primer pair forward: 5′-TGGAATTGACTTGGACCATAATAGA-3′ and reverse: 5′-AATAGTTGCTGTATCCCT-3′. The target fragment was cloned into the *Eco*RI and *Bam*HI sites of the pCDH-CMV-Puro vector (Sigma, Beijing, China). XIAP-GFP was constructed by inserting the XIAP coding sequence (forward primer: 5′-ATGACTTTTAACAGTTTTGAAGG AT-3′ and reverse primer: 5′-ACATAACATGCCTACTATAGA-3′) into *Eco*RI and *Xho*I sites of the pCMV-C1-EGFP vector (Beyotime Biotechnology, Beijing, China).

### mRNA isolation and quantitative real-time PCR

Trizol reagent (Invitrogen, Carlsbad, CA.) was applied to extract RNA from cell lines and tissue samples. cDNA was generated by 1^st^ strand cDNA synthesis using TransStart Reverse Transcriptase M-MLV (TransGen Biotech, Beijing, China) according to the manufacturer’s instructions. Quantitative real-time PCR was performed using the BioRad CFX 96 Touch real time PCR instrument (BioRad, Hercules, CA.) and a TransStart SYBR Green supermix (TransGen Biotech, Beijing, China.). The expression of the target genes was normalized to that of tubulin. Mature miR-15b-5p and the internal control U6 were detected using miScript SYBR Green PCR kit (Qiagen, Hilden, Germany), and the analysis was performed using specific primers (Table 1). The RT-qPCR data were normalized and analyzed using the 2^−ΔΔCt^ method.

**Table 1:** Primers.

Primer name	Sequence (5′-3′)
miR-15b-5p	TAGCAGCACATCATGGTT
U6-F	CTCGCTTCGGCAGCACATATACT
U6-R	ACGCTTCACGAATTTGCGTGTC
NFκB1-F	AACAGAGAGGATTTCGTTTCCG
NFκB1-R	TTTGACCTGAGGGTAAGACTTCT
Bclxl-F	GAGCTGGTGGTTGACTTTCTC
Bclxl-R	TCCATCTCCGATTCAGTCCCT
Bcl2-F	GTCTTCGCTGCGGAGATCAT
Bcl2-R	CATTCCGATATACGCTGGGAC
XIAP-F	ACCGTGCGGTGCTTTAGTT
XIAP-R	TGCGTGGCACTATTTTCAAGATA
NFκBp65-F	ATGTGGAGATCATTGAGCAGC
NFκBp65-R	CCTGGTCCTGTGTAGCCATT
Tubulin-F	ACCTTAACCGCCTTATTAGCCA
Tubulin-R	ACATTCAGGGCTCCATCAAATC

### miRNA luciferase assay

Fragment of the 3′-UTR of *NF-κB1* and *IKK-α* and a positive control for miR-15b were synthesized by Genewiz (Beijing, China). The sequence (Fig. 4A) containing the predicted binding site downstream of the pMIR luciferase reporter was then cloned to generate pMIR-NFκB1-3′UTR, pMIR-IKKα-3′UTR, and pMIR-positive control vectors, respectively. Briefly, the two reporter plasmids, Renilla luciferase plasmids, and miR-15b-5p mimics, inhibitor, or negative control (NC) were transfected into HEK293T cells at 90% confluence in 24-well plates. At 24 hours after transfection, cells were lysed and luciferase activity was assayed using the Dual-Luciferase Reporter Assay System (Promega, Madison, WI.).

### Cell viability assay

To assess the effect of miR-15b-5p on sensitivity to chemotherapy, cells were transfected with miR-15b-5p mimics or a negative control. At 8 hours post-transfection, colon cancer cells (5 × 10^3^) were initially seeded into 96-well plates. After a further 12 hours of incubation, cells were incubated with 5-FU. After 48 hours of 5-FU treatment, quantitative detection of ATP was performed using the CellTiter-Glo Luminescent Cell Viability Assay (Promega, Madison, WI) according to the manufacturer’s instructions. This method is based on the measurement of ATP production in the cells, which is proportional to the number of viable cells and is detected by means of a luciferin-luciferase reaction.

Cell viability was determined using the Cell Counting Kit-8 (Dojindo Molecular Technologies, Kumamoto, Japan) according to the manufacturer’s instructions. Cells (5 × 10^3^ cells/mL, 100 μL) were seeded into a 96-well plate. When the cells were 80–90% confluent, they were treated with 5-FU. After treatment for 24, 48, or 72 hours, 10 μL of CCK-8 solution was added to the cells, which were then incubated for another 2–3 hours protected from light. Absorbance (450 nm) was finally measured using a microplate reader.

### Apoptosis assays

Flow cytometry was used to assess apoptosis levels by staining cells with AnnexinV-FITC and propidium iodide (PI; Dojindo Molecular Technologies, Kumamoto, Japan). Colon ctrl/miR-15b OE cells were seeded into 12-well plates. After 24 hours, the anti-tumor drug (5-FU) was added. After 48 hours, cells were collected and resuspended. Double staining of cells with Annexin V-FITC and PI was used for identification of different cell populations as follows: live (FITC− PI−), early apoptotic (FITC+PI−), late apoptotic (FITC+ PI+), and necrotic (FITC−PI+) cells.

### Western blot analysis

Cells were washed with phosphate-buffered saline (PBS) and lysed in RIPA lysis buffer (Merck, Shanghai, China.). A BCA protein assay was used to standardize protein concentrations. Proteins were separated in 10–15% SDS polyacrylamide denaturing gels before being transferred to PVDF membranes. The membranes were incubated with primary antibodies at 4 °C overnight and then with the corresponding secondary antibodies at 23 °C 2 hours. The membranes were visualized by ECL. The primary antibodies used were anti-bcl-xl, -bcl-2, and -cleaved caspase 3 (Cell Signaling Technology, Danvers, MA); anti-XIAP (Upstate, OH, USA.), anti-NF-κB, -p65, and -actin (Santa Cruz Biotechnology, Santa Cruz, CA); anti-NF-κB1 (Proteintech, Wuhan, China.); and anti-IKK-α (Wanleibio, Changchun, China.). The secondary antibodies used were anti-rabbit or anti-mouse (ZSGB-BIO ORIGENE, Beijing, China) and ECL Plus (Beyotime Biotechnology, Haimen, China).

### ChIP Assays

Human embryonic kidney 293T cells (3 × 10^6^) were seeded in 100 mm dishes and cultured in the growth medium till 70% confluence. After 1% formaldehyde treatment, cells were lysed by using 600 μl lysis buffer (50 mM Tris-HCl pH 8.0, 150 mM NaCl, 5 mM EDTA, 1% NP-40, 0.5% deoxycholate, and protease inhibitors). Genome DNA was isolated and sheared into 200–1000 bp fragments with sonication. After centrifugation, the supernatants were taken and chromatin was incubated and precipitated with antibodies p65, or IgG at 4 °C overnight. Then the immune complexes were precipitated with protein A/G-Sepharose beads (GE healthcare, Beijing, China) for 4 h. After that the beads were collected after washing with lysis buffer, followed by high salt washing buffer (20 mM Tris-HCl pH 8.1, 500 mM NaCl, 1% Triton X-100, 0.1% SDS and 2 mM EDTA), LiCl washing buffer (10 mM Tris-HCl pH 8.1, 0.25 M LiCl, 1% NP-40, 1% deoxycholate and 1 mM EDTA) and TE washing buffer (10 mM Tris-HCl pH 8.1,1 mM EDTA and 4% Protease K). The immuneprecipitates were eluted by 500 μl elution buffer (1% SDS and 0.1 M NaHCO3) at 65 °C overnight. The *XIAP* promoter primers^38^ (forward primer: 5′-TGCCTGCTTAAATATTACTTTCCTCAAAA-3′, reverse primer: 5′-ACTACACGACCGCTAAGAAACATTCT-3′) were used to amplify the binding sites of p65.

### Animal experiments

Four-week-old female athymic nude mice (Charles River Laboratories, Shanghai, China) were housed under controlled light conditions and were allowed to feed ad libitum. Xenograft tumors were generated by subcutaneous injection of 4 × 10^6^ SW620/vector or SW620/15b OE cells. Tumor size was measured with linear calipers every 2 days. Tumor volume (V) was calculated using the following formula: length × width^2^/2. Once the average tumor volume reached 1000 mm^3^, animals were treated with 5-FU (20 mg/kg) once every 2 days. The mice were injected intraperitoneally daily for 24 days before they were euthanized.

A mouse colitis-associated colon cancer (CAC) model was established according to a previously published protocol^39^. BALB/c female mice were treated for 6 weeks with two drugs: dextran sulfate sodium salt (DSS, MP Biomedicals, Santa Ana, CA) and azoxymethane (AOM, Sigma–Aldrich, Milwaukee, WI). Briefly, in the CAC group, mice were intraperitoneally injected with 12.5 mg/kg AOM on day 1, after which, 2.5% DSS was included in the drinking water of the animals for 5 days, followed by 14 days of regular water. This cycle was repeated three times. The control group was given a regular diet and water. The mice in both groups were sacrificed on day 100.

All proposals were approved and supervised by the institutional animal care and use committee of Harbin Medical University. All animal studies were conducted in accordance with the National Institutes of Health guidelines for the Care and Use of Laboratory Animals.

### Statistical analysis

Numerical data are expressed as the mean ± standard deviation (SD). The difference between means was analyzed using Student’s *t*-test and differences with *p* < 0.05 were considered statistically significant.

## Electronic supplementary material

Supplementary Information
