# Supplementary material for: miR-15b-5p resensitizes colon cancer cells to 5-fluorouracil by promoting apoptosis via the NF-κB/XIAP axis
Source: Sci Rep. 2017 Jun 23;7:4194. doi: 10.1038/s41598-017-04172-z (PMC5482850; doi:10.1038/s41598-017-04172-z)
Supplement: Supplementary file 1 — Supplementary Information [file 41598_2017_4172_MOESM1_ESM.pdf]

miR-15b-5p resensitizes colon cancer cells to 5-fluorouracil by promoting apoptosis via the NF-κB/XIAP axis

Ci Zhao,[1,2] Qi Zhao,[1,2] Chunhui Zhang,[1] Guangyu Wang,[1] Yuanfei Yao,[1] Xiaoyi Huang,[2,3] Fei Zhan,[1] Yuanyuan Zhu,[1] Jiaqi Shi,[1] Jianan Chen,[1] Feihu Yan,[1] Yanqiao Zhang.[1]

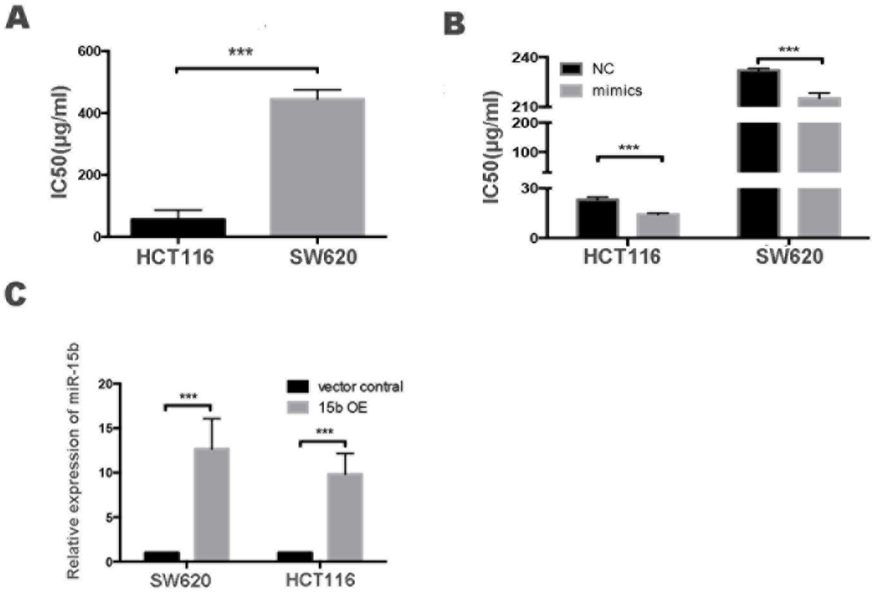

Figure S1. IC$_{50}$. (A) IC$_{50}$s of 5-Fu in SW620 and HCT116 cells based on the Luminescent Cell Viability Assay data after 48 hours of 5-Fu treatment (p <0.0001). (B) The IC$_{50}$s of mimic groups compared with those of NC groups in HCT116 (p =0.0008) and SW620 (p =0.0002) cells. (C) The expression of miR-15b-5p was assessed by RT-qPCR in SW620/15b OE and HCT116/15b OE cells (*p <0.05, **p <0.01, ***p <0.001).

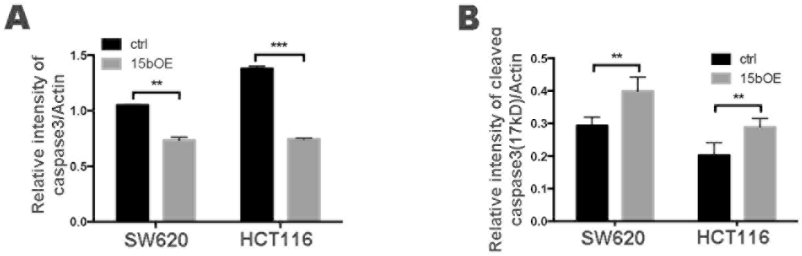

Figure S2. **Densitometric analysis of Figure 3C.** Caspase 3 (A) and cleaved caspase 3(17kD) (B) levels increased in miR-15b OE cells treated with 5-Fu. Semi-quantitative data from densitometric analysis are presented as relative ratios of each protein to actin.

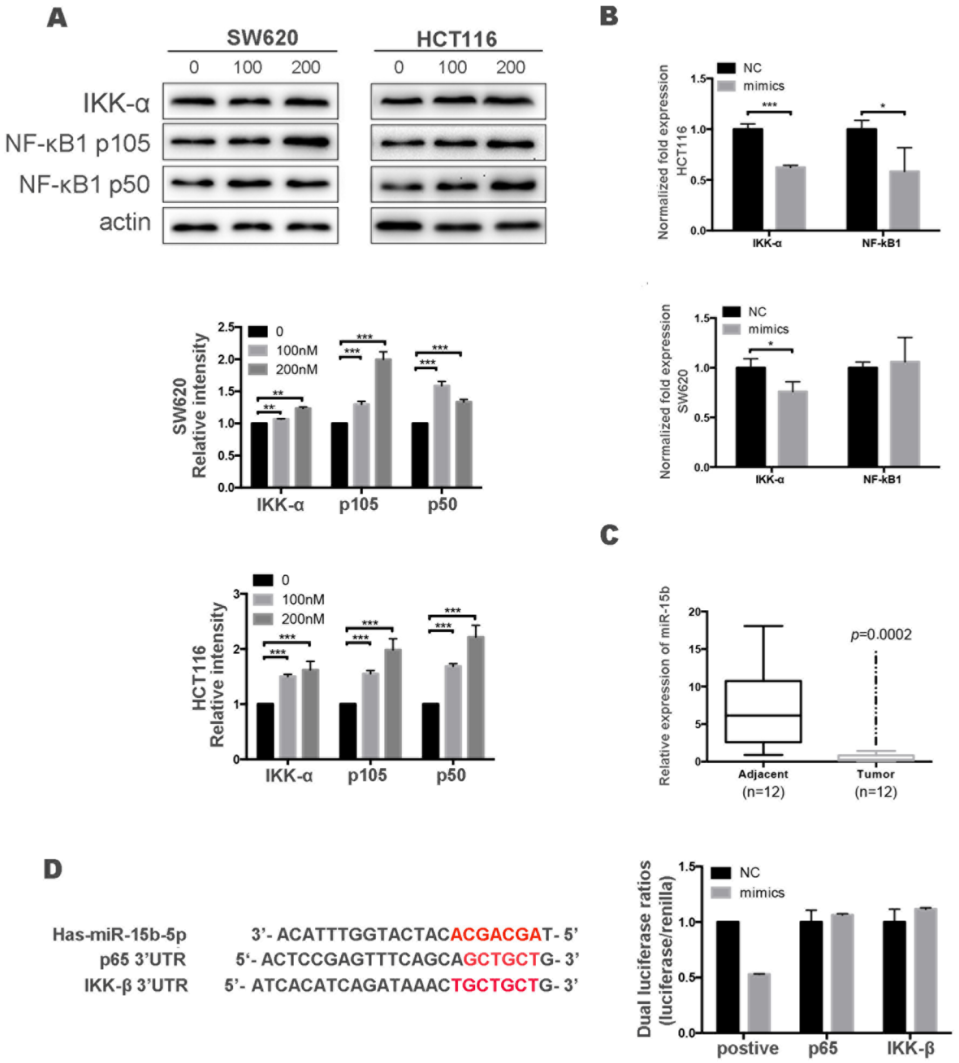

Figure S3. The supplement figure for Figure4. (A) miR-15b-5p inhibitors can increase the protein expression levels of NF-κB1 and IKK-α, and the effect was dose-dependent. (B) A scheme showing the miR-15b-5p putative binding sites in the 3′-UTR of *p65* or *IKK-β* mRNA. miR-15b-5p mimic and the pMIR-p65-3′UTR or pMIR-IKKβ-3′UTR vector was co-transfected with in HEK293T cells. The relative luciferase activity was measured. (C) The expression levels of *NF-κB1* and *IKK-α* mRNA were assessed by RT-qPCR in SW620 and HCT116 cells. (D) The expression of miR-15b-5p was assessed by RT-qPCR in 12 pairs of primary colorectal cancer tissues and their adjacent normal tissues.

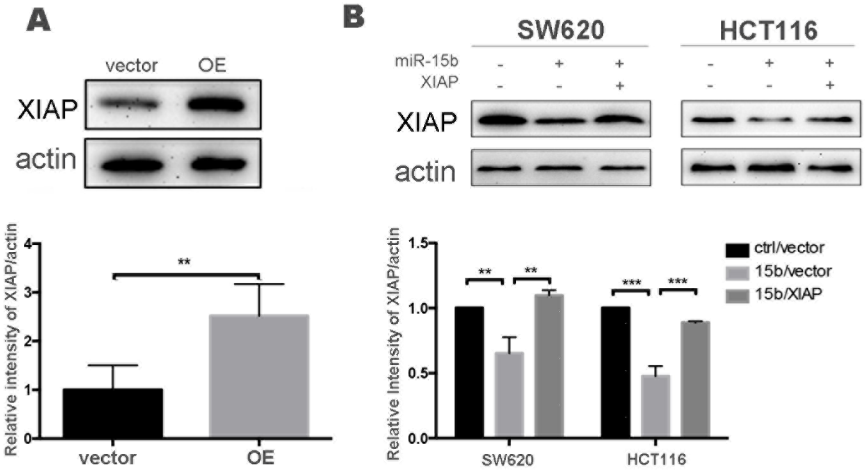

Figure S4. **The protein expression of XIAP and semi-quantitative analysis**. (A) Expression of XIAP after recombinant plasmid transfection were assessed by western blotting in HEK293T cells. (B) Expression of XIAP after co-transfection with the miR-15b-5p and *XIAP* expression vectors before 5-FU treatment in SW620 or HCT116 cells. Three such experiments were quantified by measuring the intensity of XIAP relative to the Actin (loading) control.
